# Comparative analysis of the pregnancy rate via *in vitro* fertilization vs. previous artificial insemination in patients with unexplained infertility

**DOI:** 10.5935/1518-0557.20210038

**Published:** 2022

**Authors:** Maitane Alonso de Mendieta, Alejandro Sánchez Aranda, Juan Francisco Molina López, Armando Miguel Roque Sánchez, Esperanza Carballo Mondragón, Leonor Ángela Durán Monterrosas, Alberto Kably Ambe

**Affiliations:** 1 Centro Mexicano de Fertilidad Dr. Alberto Kably, Mexico State, Mexico; 2 The American British Cowdray Medical Center, Mexico City, Mexico

**Keywords:** *In vitro* fertilization, intrauterine insemination, unexplained infertility

## Abstract

**Objective:**

To compare the clinical pregnancy rate among patients undergoing direct in vitro fertilization vs. in vitro fertilization after two cycles of intrauterine insemination in couples with unexplained infertility.

**Methods:**

Comparative cross-sectional, retrospective study from 2016 to 2019, from the Centro Mexicano de Fertilidad Doctor Alberto Kably. The patients with unexplained infertility were divided into two groups, direct in vitro fertilization and a group of in vitro fertilization after intrauterine insemination, and we compared the rate of pregnancy and live births in both cases.

**Results:**

89 couples with unexplained infertility were included, the in vitro fertilization after intrauterine insemination group (n=46) and direct in vitro fertilization group (n=43). The direct in vitro fertilization group resulted in a higher clinical pregnancy rate throughout the study compared to the other group (55.8% *vs*. 34.8%, OR 2.37; 95% CI 1.008 - 5.57, *p*=0.046). However, there was no difference in the rate of live newborns (*p*=0.12). When analyzing the data by cycle, we noticed a statistical difference in both, the clinical pregnancy rate in the direct in vitro fertilization group (38.7% *vs*. 16.7%, OR 3.2; 95% CI 1.50-6.62), as well as the rate of live newborns (32.3 % *vs*. 14.6%, OR 2.79; 95% CI 1.28-6.07, *p*=0.008).

**Conclusions:**

In the *in vitro* fertilization group, as first-line treatment for unexplained infertility, the patients had a higher pregnancy rate.

## INTRODUCTION

Unexplained infertility is the inability to achieve a pregnancy after 12 months of regular unprotected sexual intercourse in patients under 35 years of age or after 6 months in patients over 35 years of age with adequate ovarian, tubal, uterine and cervical function, as well as adequate testicular function (Zegers-Hochschild *et al*., 2017). About 30% of couples with infertility are diagnosed with unexplained infertility (Practice Committee of the American Society for Reproductive Medicine, 2020; Kably Ambe *et al.*, 2012). The Canadian Society for Infertility and Andrology reports that unexplained infertility rates can reach up to 50% (Buckett & Sierra, 2019).

Most of the treatments described for unexplained infertility initially consist of expectant management, followed by intrauterine insemination (IUI) with or without controlled ovarian hyperstimulation (COH). However, there is a lack of evidence regarding the effectiveness of such treatment (Scholten *et al*., 2017).

Intrauterine insemination is considered better than expectant management, since it increases the number of oocytes available for fertilization thanks to controlled ovarian hyperstimulation and an increase in the number of motile sperm within the female reproductive system at the time of ovulation (Practice Committee of the American Society for Reproductive Medicine, 2020).

In 2014, the NICE (National Institute for Health and Care Excellence of the United Kingdom) guidelines recommended not performing insemination or stimulation with oral medications due to lack of effectiveness, and in case of not being successful after a year of regular unprotected sexual intercourse, it is recommended to switch immediately to in vitro fertilization (O'Flynn, 2014).

The *Consenso Nacional Mexicano de Reproducción Asistida* [Mexican National Consensus on Assisted Reproduction], published in 2012, refers that the evidence suggested in vitro fertilization is more successful for the treatment of unexplained infertility compared to IUI with COH [Controlled Ovarian Hyperstimulation, HOC, for its initials in Spanish]. However, at that time there were no differences between IVF and expectant management. Finally, the consensus recommendation was to perform the most basic treatments such as expectant management or IUI prior to IVF due to the high costs and risks of complications of the latter (Kably Ambe *et al*., 2012).

Considering these recommendations, the debate persists over which is the best moment to migrate to the IIU or IIU with COH to a highly complex technique such as in vitro fertilization (IVF). In a prospective study carried out by Smith *et al*. (2011), they reported that in couples after three intrauterine inseminations (IUI) there was no higher rate of pregnancies in subsequent IUI, for which reason it is recommended to switch to highly complex techniques.

The American Society of Reproductive Medicine (ASRM) recommends intrauterine insemination as long as it is accompanied by controlled ovarian stimulation either with oral agents or with low-dose gonadotropins. If the couple does not achieve pregnancy after several IUIs with COH, performing an IVF should follow. On the other hand, they also report that the couple can be immediately transferred to IVF to reduce emotional and physical stress, since this can shorten the time to achieve pregnancy (Practice Committee of the American Society for Reproductive Medicine, 2020). There are studies such as the FASTT study carried out by Reindollar *et al*. (2010), who compared couples with a diagnosis of unexplained infertility that did not achieve pregnancy after a cycle of IUI with clomiphene citrate (CC), and immediately switched to IVF with a pregnancy rate of 32.7% *vs*. patients who continued with IUI with different types of ovarian hyperstimulation controlled with clomiphene citrate or gonadotropins, with pregnancy rates of 7.6% and 9.8%, respectively. They also reported lower costs in the immediate IVF group compared to the other two groups (Reindollar *et al*., 2010).

The FASTT study concluded that young patients with unexplained infertility do not benefit from IUI with gonadotropins. However, in this study they did not document if this strategy also works in couples who are at the end of their reproductive life. In 2014, a FORT-T clinical trial was published, aiming to determine if in vitro fertilization is the optimal treatment for patients of advanced reproductive age with unexplained infertility, in order to shorten the time of conception. In this study, the population had to be between 38-42 years old and was divided into three groups, IUI with CC, IUI with rFSH [Follicle Stimulating Hormone], and finally the immediate IVF group. If the patients did not achieve pregnancy after two cycles of IUI, they were switched to IVF. The total study population was 154 couples, 51 couples in IUI with CC, 52 couples in IUI with rFSH, and finally, 51 couples in immediate IVF. In the first two cycles the pregnancy rate was significantly higher in the immediate IVF group compared to IUI with CC and IUI with rFSH, with 49%, 21.6% and 17.3%, respectively (*p*=0.0067). Regarding the live newborn rate, the immediate IVF group presented 31.4% *vs*. IUI with CC 15.7% and IUI with rFSH 13.5% (*p*=0.35). In conclusion of the FORT-T study, couples of advanced reproductive age benefited from directly moving to the IVF group, reducing conception time and being exposed to fewer cycles (Goldman *et al*., 2014).

The Canadian guidelines for the management of unexplained infertility (2019), recommend that in vitro fertilization can be used as a first-line treatment for unexplained infertility with a B1 level of evidence, and it should even be offered as an option for couples with unexplained infertility who have already undergone 3 cycles of IUI with COH without success, with an A1 level of evidence (Buckett & Sierra, 2019).

## MATERIALS AND METHODS

This is a cross-sectional, comparative, retrospective study with two study groups. The sample calculation was carried out according to the model proposed by Kelsey *et al*. (1996) for a 31.7% difference in proportions between exposed and unexposed cases (Goldman *et al*., 2014), with a significance level of 95% and a power of 80%, with which a total sample size of 80 couples was obtained, 40 for the IUI + IVF group and 40 for the dIVF group.

For the univariate analysis according to their distribution, continuous variables were reported with means and standard deviation for normal distribution (eg. Age) or with medians and ranges for non-parametric distribution, qualitative variables were reported as proportions (eg. Pregnancy rate). For the bivariate analysis, the comparison of continuous variables with normal distribution between two unrelated groups used the unpaired t-student test, and between dichotomous qualitative variables of two groups, the Chi-2 or U of Mann-Whitney test was performed for three groups. The multivariate analysis was performed with a binary logistic regression, adjusting for the variables of the basal state, main maneuver and peripheral for the dichotomous outcome of clinical pregnancy. Odds ratio and 95% confidence intervals were calculated and a *p-*value less than 0.05 was considered statistically significant.

### Selected Population

The population belongs to couples with a diagnosis of unexplained infertility treated at the *Centro Mexicano de Fertilidad Doctor Alberto Kably* [Mexican Fertility Center Doctor Alberto Kably] in the state of Mexico from January 2016 to December 2019 that met the inclusion criteria. The eligibility criteria were patients treated at the *Centro Mexicano de Fertilidad Doctor Alberto Kably Ambe* [Mexican Fertility Center Doctor Alberto Kably Ambe] who had a proven unexplained infertility diagnosis with adequate ovarian function with FSH<10UI/mL levels, adequate antral follicular count and/or a (HAM) ≥1.1 ng/mL antimullerian hormone, presence of ovulatory cycles, proven patency of at least one tube by hysterosalpingography or by laparoscopy, normal values of thyroid stimulating hormone (TSH) and prolactin (PRL) and normal male factor; with adequate density, progressive mobility and morphology, in case of presenting mild male factor with morphology of 3%, they had to have a normal DNA fragmentation index to be included in this study.

The clinical pregnancy was confirmed with a positive beta fraction and later a transvaginal ultrasound with the presence of a gestational sac, embryonic pole and fetal heart rate.

### Protocol

The included couples were divided into 2 groups, the IUI + IVF *vs*. the dIVF. In the IUI + IVF group, the patients underwent two cycles of intrauterine insemination with ovarian hyperstimulation controlled with gonadotropins or menotropins, and in case of not presenting pregnancy, they later switched to an *in vitro* fertilization, while in the dIVF group, the patients underwent *in vitro* fertilization directly.

## RESULTS

**Figure 1:**
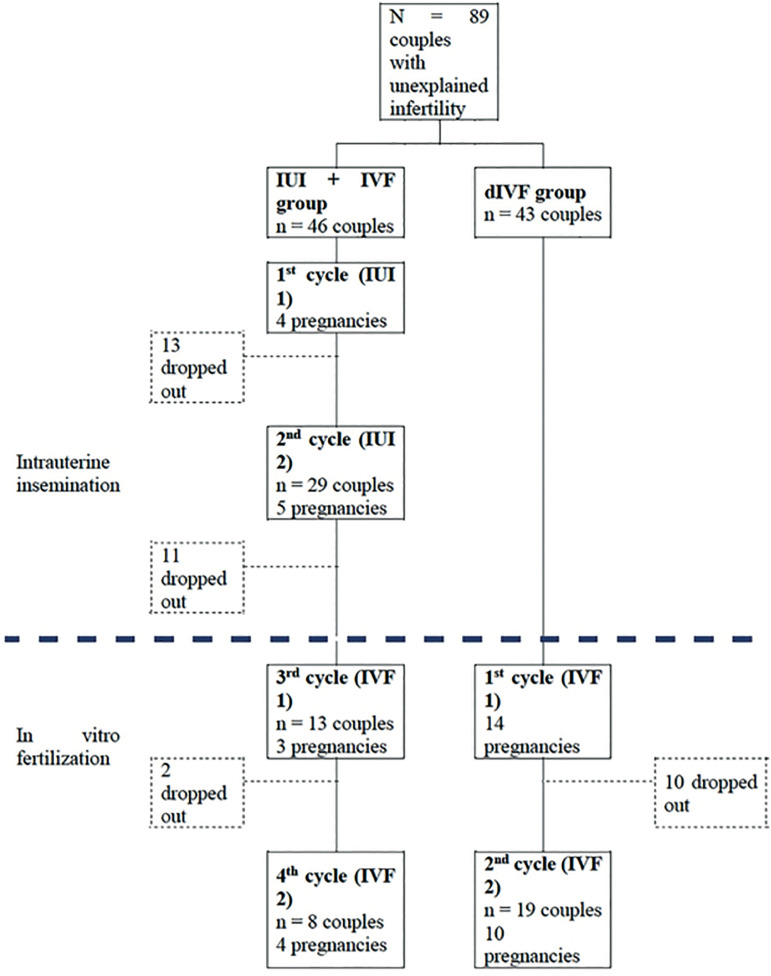
Study diagram. IUI: intrauterine insemination, dIVF: direct *in vitro* fertilization, IVF: *in vitro* fertilization.

Eighty-nine couples were included in the two groups. The first group composed of IUI + IVF (n=46), and the second group of couples undergoing IVF (n=43). In the IUI + IVF group, there were 16 clinical pregnancies (34.8%), nine of which were in insemination cycles and seven in IVF; 26 couples did not complete the study, which corresponds to a dropout rate of 56.5%. While in the second group of dIVF, 24 (55.8%) clinical pregnancies were reported, 10 couples dropped out (23.3%) (Figure 1).

We compared the demographic data between both groups; the dIVF group had an older age (36.2 *vs*. 34.1 years, *p*=0.019) and less time of infertility (less than one year, *p*=0.028), compared to the IUI + IVF group. The results of the type of infertility, seminogram and basal hormonal profile did not show significant difference (Table 1).

**Table 1:** Demographic characteristics of patients with unexplained infertility.

	IIU+IVF n=46	IVF n=43	*p*-value
Female age (years)	34.1±4.27	36.2±4.08	0.019
Male age (years)	36.4±4.09	38.1±4.21	0.067
Type of infertility n (%)			0.78
Primary	27 (58.7)	24 (55.8)	
Secondary	19 (41.3)	19 (44.2)	
Time of infertility n (%)			0.028
< 1 year	8 (17.4)	8 (17.4)	
From 1 - 2 years	24 (52.2)	24 (52.2)	
> 3 years	14 (30.4)	14 (30.4)	
FSH (mUI/ml)	6.07±2.46	5.79±1.40	0.51
LH** (mUI/ml)	4.68 (0.7-17.3)	5 (2.5-12.3)	0.42
Estradiol (pg/ml)	38.7±15.6	39.2±14.2	0.89
Prolactin (ng/dl)	14.9±5.94	14.3±5.57	0.62
AMH** (ng/ml)	2.58 (1.04-16)	2.12 (1.10-12.3)	0.14
TSH (mUI/L)	1.59±0.51	1.62±0.6	0.79
AFC** (n)	8 (4 - 25)	8 (3 - 23)	0.80
Seminogram **			
Density (mill/ml)	80 (32 - 288)	84 (42 - 150)	0.43
Progressive motility (%)	58 (32 - 92)	61 (30 - 76)	0.10
Morphology (%)	4 (3 - 9)	5 (3 - 7)	0.71

IUI + IVF: intrauterine insemination and in vitro fertilization, IUI: intrauterine insemination, IVF: in vitro fertilization, FSH: Follicle-stimulating hormone, LH: Luteinizing Hormone, HAM: Antimullerian hormone, TSH: Thyroid-stimulating hormone, AFC: antral follicle count.

** Results represented in median (range). The rest was calculated with mean ± standard deviation.

A total of 158 cycles were reported in the study, within the IUI + IVF group, 96 cycles were performed, 75 of them correspond to intrauterine insemination cycles and 21 to IVF; 9 pregnancies resulted from the 75 cycles of insemination, while the remaining 7 pregnancies were the result of cycles performed with IVF. On the other hand, in the dIVF group, 24 pregnancies were achieved out of 62 cycles performed.

Within the IUI + IVF group, three different ovarian stimulation schemes were used (menotropins, rFSH / rLH and rFSH monotherapy), while in the dIVF group only two schemes were used (menotropins and rFSH / rLH). The menotropins scheme was used in 69 cycles (32 cycles in the IUI + IVF group and 37 cycles in the dIVF group), obtaining 18 pregnancies (26.1%) and 14 live newborns (20.3%) in both groups. While the scheme of recombinant gonadotropins (rFSH / rLH) was used in 37 cycles (12 in the IUI + IVF group and 25 cycles in dIVF) obtaining 17 pregnancies (45.9%) and 15 live newborns (40.5%) in both groups. rFSH monotherapy was used in 52 cycles only in the IUI + IVF group, which resulted in 5 clinical pregnancies (9.6%) and 5 live newborns (9.6%) (Table 2).

**Table 2:** Association of ovarian stimulation scheme with number of clinical pregnancies and live newborns by number of cycles.

Stimulation scheme	No. of clinical pregnancies / No. of cycles (%)			No. of live newborns / No. of cycles (%)		
	IIU + FIV (n=96 cycles)	dFIV (n=62 cycles)	Total (n=158 cycles)	IIU + FIV (n=96 cycles)	dFIV (n=62 cycles)	Total (n=158 cycles)
Menotropins (hMG) (n=69 cycles)	6/32 (18.8)	12/37 (32.4)	18/69 (26.1)	4/32 (12.5)	10/37 (27)	14/69 (20.3)
rFSH (n=52 cycles)	5/52 (9.6)	--	5 /52 (9.6)*	5/52 (9.6)	--	5/52 (9.6)**
rFSH/rLH (n=37 cycles)	5/12 (41.7)	12/25 (48)	17/37 (45.9)*	5/12 (41.7)	10/25 (40)	15/37 (40.5)**

* In the intergroup analysis it showed a significant difference (p<0.001).

** In the intergroup analysis it showed a significant difference (p=0.002).

In the intergroup analysis, the rFSH/rLH scheme showed a higher rate of pregnancies (45.9% *vs*. 9.6%, *p*<0.001) and live newborns (40.5% *vs*. 9.6%, *p*=0.002) when compared with the monotherapy scheme of rFSH. There were no differences in the rate of pregnancy or live births between the rFSH *vs*. Menotropins groups, as well as rFSH/rLH *vs*. Menotropins.

The dIVF group resulted in a higher clinical pregnancy rates throughout the study compared to the IUI + IVF group (55.8% *vs*. 34.8%, OR 2.37; 95% CI 1.008 - 5.57, *p*=0.046). However, there was no difference in the live birth rate (*p*=0.12). However, if we analyze the data by cycle, we can see a statistical difference both in the clinical pregnancy rate in the IVF group (38.7% vs. 16.7%, OR 3.2; 95% CI 1.50-6.62, *p*=0.002), as well as in the rate of live newborns (32.3% vs. 14.6%, OR 2.79; 95% CI 1.28-6.07, *p*=0.008) (Table 3).

**Table 3:** Number and rate of clinical pregnancies and live newborns per group.

	Number of couples who started treatment	Number of cycles started	Clinical pregnancy n (%)			Number of live newborns n (%)		
			No.	Per cycle	Per Couple	No.	Per cycle	Per couple
IUI+IVF								
-IUI	46	75	9	12%^e^	19.6%	8	10.7%^f^	17.4%
-IVF	13	21	7	33.3%^e^	53.8%	6	28.6%^f^	46.6%
-Total		96	16	16.7%^a^	34.8%^b^	14	14.6%^c^	30.4%^d^
dIVF n=43	43	62	24	38.7%^a^	55.8%^b^	20	32.3%^c^	46.5%^d^

IUI + IVF: intrauterine insemination and *in vitro* fertilization, IVFd: direct in vitro fertilization, OR: odds ratio, CI: confidence interval.

a Comparison of the IUI + IVF group vs. dIVF per cycle with *p*-value=0.002, OR 3.2 (1.50-6.62).

b Comparison of the IUI + IVF group vs. dIVF per couple with *p*-value=0.046, RM 2.37 (1.008- 5.57).

c Comparison of the IUI + IVF group vs. dIVF per cycle with *p*-value=0.008, OR 2.79 (1.28-6.07).

d Comparison of the IUI + IVF group vs. dIVF per couple with *p*-value = 0.12, RM 1.98 (0.83-4.73).

e Comparison of IUI vs IVF cycles in the IUI + IVF group with *p*-value = 0.020, RM 3.66 (1.17-11.51).

f Comparison of IUI vs IVF cycles in IUI + IVF group with *p*-value = 0.040, RM 3.35 (1.01-11.09).

Of the couples that did not continue with the study, there were four spontaneous pregnancies within 6 months of discontinuation of the protocol. Three were from the IUI + IVF group (0.7%) and one from the dIVF group (0.2%).

Regarding adverse events, there was no case of severe ovarian hyperstimulation syndrome reported. 6 twin pregnancies (15%) were reported, 2 in the IUI + IVF group, one of which was intrauterine insemination and the second with IVF technique, which corresponds to 12.5% and 4 in the dIVF group (16.7%) without presenting significant difference (*p*=0.718).

## DISCUSSION

Unexplained infertility is diagnosed in about 30% of infertile couples worldwide; however, until now there has not been a global consensus about the optimal initial management for these patients (Practice Committee of the American Society for Reproductive Medicine, 2020). This demonstrates a heterogeneity regarding the handling of these couples out of a non-negligible number.

In our study, there as a higher pregnancy rate in the group of patients with dIVF, compared to the IUI + IVF group (55.8% *vs*. 34.8%, OR 2.37; 95% CI 1.008-5.57, *p*=0.04). However, there was no difference when comparing the live newborn rate of 46.5% *vs*. 30.4%, (OR of 1.98 [95% CI 0.83-4.73], *p*=0.12). There were similar results reported in the FORT-T study (Goldman *et al.*, 2014), no difference was observed in the live newborn rate in the dIVF group, compared to the IUI + CC and IUI + rFSH (31.4%, 15.7% and 13.5%; *p*=0.35, respectively).

On the other hand, both studies had better rates of clinical pregnancy (*p*=0.002) and live newborns per cycle (*p*=0.008) IVFd *vs*. IUI + IVF group. In the FORT-T study, 91 cycles of IUI with rFSH were performed, reporting a pregnancy rate of 7.7% per cycle, while in our study, in the IUI + IVF group, 75 cycles of IUI were performed with a pregnancy rate of 12 % per cycle. Of the couples in the IUI + IVF groups that did not achieve pregnancy after 2 cycles, they switched to IVF. In our study, we ran 21 IVF cycles with an IVF success rate for clinical pregnancy of 33.3%, this being a better rate than the one reported in the FORT-T study of 25%, maybe it is important to remark that patients in the present study belonging to the IUI + IVF group were younger than the dIVF group, which could explain the better pregnancy rates comparing to the FORT-T study.

Something that draws attention in both studies is that a large part of the pregnancies in the IUI + IVF group were due to the fact that they finally switched to IVF techniques (Goldman *et al*., 2014), since the pregnancy rate per insemination cycles is very low compared to IVF.

The guidelines for the management of couples with unexplained infertility published in 2020 by the American Society for Reproductive Medicine (ASRM) report that the literature is not yet sufficient to support in vitro fertilization as the first line therapy in the management of unexplained infertility over expectant management for 6 months or over IUI with COH in patients under 38 years of age. However, within the same guidelines they report that most of the recommendations that support the use of IUI as first-line management are based on old studies with lower rates of IVF effectiveness compared to the ones that are currently used (Practice Committee of the American Society for Reproductive Medicine, 2020). While in our study, the effectiveness of IVF over IUI in patients with unexplained infertility was evident.

Within the same guidelines, the use of direct IVF in patients over 38 years of age is recommended for achieving a higher pregnancy rate in less time, based on the FORT-T study (Practice Committee of the American Society for Reproductive Medicine, 2020; Goldman *et al*., 2014). As we noted in our study, in both groups, dIVF and IUI + IVF, most couples had been trying to achieve pregnancy for at least more than a year, regardless of the age of the patients, and a significant difference was reported in the dIVF group, in which patients were older (36.2±4.08 *vs*. 34.1±4.27, *p*=0.019), and despite this difference, there was a higher pregnancy rate in the dIVF group, proving the superiority of dIVF despite the poor prognosis factor, which was the patient's age.

In the IUI + IVF group, there were 26 couples who decided not to finish the study, with a dropout rate of 70.3%, unlike the direct IVF group, where 10 patients discontinued the study, with a dropout rate of 34.5%, although we do not really know the causes of desertion, since it is not part of the objectives of our study, it can be assumed that it was due to economic, emotional or frustration that they decided to continue their treatment in another clinic. Which suggests that these patients require more attention and improve the initial treatments offered to reduce the time to pregnancy.

Regarding the number of inseminations prior to transferring the patients to in vitro fertilization, the Canadian guidelines recommend performing three IUIs. And, in case of not presenting pregnancy, proceed to IVF with a higher level of evidence, than to transfer the patients directly to IVF (Buckett & Sierra, 2019). However, in our study performing IUI prior to IVF can increase the final cost since 80.4% of the population that underwent insemination did not become pregnant and had to finally switch to IVF.

The possible cause for the lack of recommendation for direct IVF may be due to the adverse events that occur more frequently with highly complex techniques, such as ovarian hyperstimulation syndrome and multiple pregnancies, which in recent decades has decreased thanks to the improvement in protocols and surveillance worldwide; as well as in our study where there were no reports of moderate or severe ovarian hyperstimulation syndrome, but it was reported that 15% of pregnancies were twins, without achieving significance between both groups. Although the NICE guidelines report a higher risk of multiple pregnancies in IUI with the use of gonadotropins, this is due to the fact that they perform a single embryo transfer during IVF cycles, unlike in our country, even in our clinic where the transfer of two embryos is commonly performed (O'Flynn, 2014) the provincial government of Québec, Canada introduced funding of assisted reproduction treatment through the provincial health programme. Alongside this benefit, legislation was introduced to control assisted reproduction treatment activities in the province, including restrictions on the number of embryos that could be transferred in any one cycle. The aim of the programme was to transfer a single embryo in every cycle; multiple embryos could be transferred under suboptimal conditions but required physician justification. In the first 3 months of this programme, 1353 cycles of IVF were performed in five Québec assisted reproduction centres, with an overall clinical pregnancy rate of 32% per embryo transfer and 50% of transfers used elective single-embryo transfer (eSET.

Finally, we must remember that these studies were conducted in couples with unexplained infertility, and not in couples with anovulation or other explainable causes, where the rates of pregnancy through intrauterine insemination are better than those reported in unexplained infertility.

The limitation of the study is that it is a retrospective study and it is necessary to carry out prospective studies to unify the groups and stimulation protocols to determine if dIVF is really the best management for couples with unexplained infertility.

## CONCLUSION

The in vitro fertilization group, as first-line treatment for unexplained infertility patients, demonstrated a higher pregnancy rate compared to the intrauterine and later in vitro insemination group.
